# MicroRNA Expression Profiling of Oligodendrocyte Differentiation from Human Embryonic Stem Cells

**DOI:** 10.1371/journal.pone.0010480

**Published:** 2010-05-05

**Authors:** Brian S. Letzen, Cyndi Liu, Nitish V. Thakor, John D. Gearhart, Angelo H. All, Candace L. Kerr

**Affiliations:** 1 Department of Biomedical Engineering, The Johns Hopkins University School of Medicine, Baltimore, Maryland, United States of America; 2 Department of Cell and Developmental Biology, Institute of Regenerative Medicine, University of Pennsylvania, Philadelphia, Pennsylvania, United States of America; 3 Department of Animal Biology, Institute of Regenerative Medicine, University of Pennsylvania, Philadelphia, Pennsylvania, United States of America; 4 Department of Neurology, Johns Hopkins University, Baltimore, Maryland, United States of America; 5 Department of Gynecology and Obstetrics, Institute for Cell Engineering, Johns Hopkins University, Baltimore, Maryland, United States of America; 6 Stem Cell Program, Institute for Cell Engineering, Johns Hopkins University, Baltimore, Maryland, United States of America; Universidade Federal do Rio de Janeiro (UFRJ), Brazil

## Abstract

**Background:**

Cells of the oligodendrocyte (**OL**) lineage play a vital role in the production and maintenance of myelin, a multilamellar membrane which allows for saltatory conduction along axons. These cells may provide immense therapeutic potential for lost sensory and motor function in demyelinating conditions, such as spinal cord injury, multiple sclerosis, and transverse myelitis. However, the molecular mechanisms controlling OL differentiation are largely unknown. MicroRNAs (**miRNAs**) are considered the “micromanagers” of gene expression with suggestive roles in cellular differentiation and maintenance. Although unique patterns of miRNA expression in various cell lineages have been characterized, this is the first report documenting their expression during oligodendrocyte maturation from human embryonic stem (**hES**) cells. Here, we performed a global miRNA analysis to reveal and identify characteristic patterns in the multiple stages leading to OL maturation from hES cells including those targeting factors involved in myelin production.

**Methodology/Principal Findings:**

We isolated cells from 8 stages of OL differentiation. Total RNA was subjected to miRNA profiling and validations preformed using real-time qRT-PCR. A comparison of miRNAs from our cultured OLs and OL progenitors showed significant similarities with published results from equivalent cells found in the rat and mouse central nervous system. Principal component analysis revealed four main clusters of miRNA expression corresponding to early, mid, and late progenitors, and mature OLs. These results were supported by correlation analyses between adjacent stages. Interestingly, the highest differentially-expressed miRNAs demonstrated a similar pattern of expression throughout all stages of differentiation, suggesting that they potentially regulate a common target or set of targets in this process. The predicted targets of these miRNAs include those with known or suspected roles in oligodendrocyte development and myelination including C11Orf9, CLDN11, MYTL1, MBOP, MPZL2, and DDR1.

**Conclusions/Significance:**

We demonstrate miRNA profiles during distinct stages in oligodendroglial differentiation that may provide key markers of OL maturation. Our results reveal pronounced trends in miRNA expression and their potential mRNA target interactions that could provide valuable insight into the molecular mechanisms of differentiation.

## Introduction

Oligodendrocytes (**OLs**) play a critical role within the central nervous system (**CNS**) by producing the insulating protein myelin, which provides efficient neuronal conductivity by decreasing ion leakage and capacitance of axonal membranes. Significant damage to OLs results in demyelination and hinders effective communication among neurons. Correspondingly, CNS demyelinating conditions, such as spinal cord injury, multiple sclerosis, transverse myelitis, and optic neuritis can result in severe motor, sensory, and cognitive impairment [1], [2]. Cellular replacement strategies involving the transplantation of cells from the oligodendrocyte lineage to induce remyelination have been utilized in various studies [3], [4], [5], [6]. One of the most promising sources for this cell therapy is human embryonic stem (**ES**) cells, which are capable of unlimited proliferation and differentiation into cell types from all three germ layers. As such, human ES cell-derived approaches for producing oligodendrocyte cells offer significant potential for these therapeutic efforts. The utility of hES-derived OL progenitors (**OPCs**) have been shown in animal models of multiple sclerosis and spinal cord injury [3], [6], [7], [8]. However, little is known about the fundamental regulatory mechanisms that control the differentiation of human ES cells into OLs and only a handful of laboratories have demonstrated the ability to derive mature OLs [6], [9]. The difficulty in deriving these cells is due in part to the lack of knowledge regarding the regulators of oligodendrocyte development.

MicroRNAs (miRNAs) are ∼23 nucleotide molecules that have been termed the “micromanagers” of gene expression [10]. They have been widely shown to regulate protein expression at the posttranscriptional level by binding to the mRNA of protein-coding genes. Specifically, the “seed” region of a miRNA (centered on nucleotides 2–7) binds to the 3′ untranslated region (UTR) of target mRNAs via Watson-Crick complementary base pairing. Subsequently, these target mRNAs are most commonly repressed by undergoing Argonaute-catalyzed cleavage and/or destabilization [11], [12]. MiRNAs are known to play important roles in many cellular processes [13], [14], [15], including stem cell maintenance [16] and differentiation including those of the neural lineage [17], [18]. However, only a few laboratories have investigated the specific miRNA expression in murine oligodendrocytes [19], [20], [21] and no study to date has characterized miRNA expression in human OLs. This information is critical since miRNAs are potential key regulators of differentiation, as demonstrated by their dynamic expression throughout development. In fact, miRNA deficient mice with Dicer deletions were shown to display significant impairment in oligodendrocyte differentiation and production of myelin genes [22], [23].

Using a human ES cell derived model of OL differentiation, we have shown that miRNAs are temporally regulated during this process by studying neural embryoid body (**EB**) cells, neural progenitor (**NP**) cells, glial-restricted precursor (**GP**) cells, oligodendrocyte precursor (**OP**) cells, and OLs. Additionally, this analysis reveals a pronounced relationship between the highest differentially expressed miRNAs at a particular stage and their corresponding grouped expression over the course of oligodendrocyte differentiation. These results suggest the possibility that they share common targets. Moreover, a number of these miRNAs have putative targets in proteins with known or suspected roles in myelination. Thus, the following study is the first report performing a thorough characterization of global miRNA expression levels encompassing the complete differentiation scheme from hES cells to terminally differentiated OLs.

## Results

### Embryonic stem cell differentiation into oligodendrocyte lineage cells

**Figure 1:**
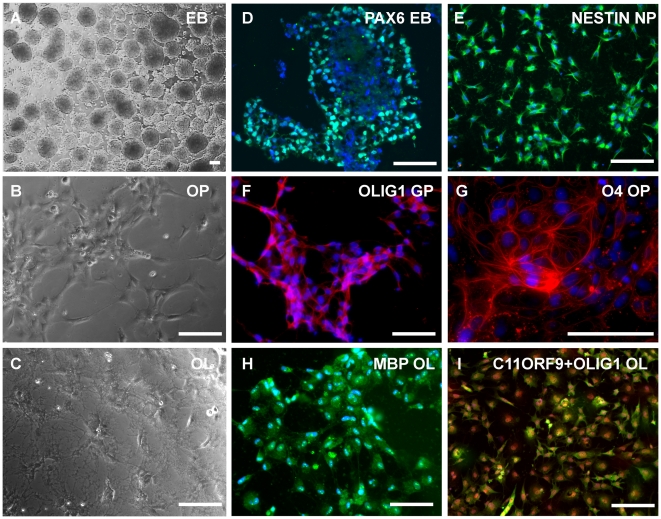
Morphological development and indirect immunofluorescence detection of key markers of OLs in culture. (a) Neural EBs derived from hES cells. (b) Immature OPCs derived from hES cells demonstrate bipolar morphology, (c) while later progenitors develop multiple oligodendrocytic projections. (d) Pax6 (green) in cryosectioned EBs after 15 days, (e) A2B5 positive sorted NPs expressing nestin (green), (f) Olig1 (red) expressing GPs, (g) O4+ (red) OP cells, and (h) MBP (green) expressing OLs which also express (i) C11orf9 (human homolog to MRF) red nuclei and Olig1 (green). Dapi (blue) stain nuclei. Scale bars: 100 um.

The differentiation of human ES cells into OL lineage cells has been previously demonstrated by a handful of laboratories. Recently, our group has implemented a modified differentiation protocol demonstrating increased purity and efficiency [24], with >95% of cells expressing appropriate markers at defined cell stages. Specific stages were defined in culture by changes in growth factors, substrate adherence conditions, morphology (**Figure 1a–c**), and gene expression of known markers (**Figure 1d–i**). Neural differentiation was initiated by EB formation as commonly used to generate early neuroectodermal cells transitioning from undifferentiated ES cells. In suspension, these cells begin to lose expression of key markers of pluripotency such as OCT4 and NANOG and begin to express SOX1, PAX6, and NESTIN. Once plated these cells begin a flattened morphology and divide as a monolayer in the dish. These cells continue to express early neural markers but also begin to express A2B5 and Sox10. For the purpose of this study, these cells represent early to late neural progenitor formation, respectively. Glial progenitors produce a bipolar morphology and begin to express Olig1, PDGFRα and NG2. Upon further culture and the addition of PDGF-AA, GPs begin to exhibit multiple filopodial extensions and they begin to express O4 and later express O1, GalC and CNPase markers [25], [26], [27], [28], [29], [30], [31]. These OP cells were evaluated over time which we define as early, mid- and late OP cells. Specifically, cells at the early OP stage began to express O4, while cells of the mid OP stage expressed O1 and GalC, and the late OP stage expressed CNPase. Although these are commonly known as markers of mature oligodendrocytes, these cells did not express myelin basic protein (**MBP**). Moreover, these cells continued to proliferate in culture, indicative of a progenitor-like state. The production of MBP after the addition of T3 and withdrawal of growth factors corresponded with their maturation into OLs. This was consistent with changes in their morphology with large cell bodies and extensive filopodial branching.

### MiRNA Profiles

**Figure 2:**
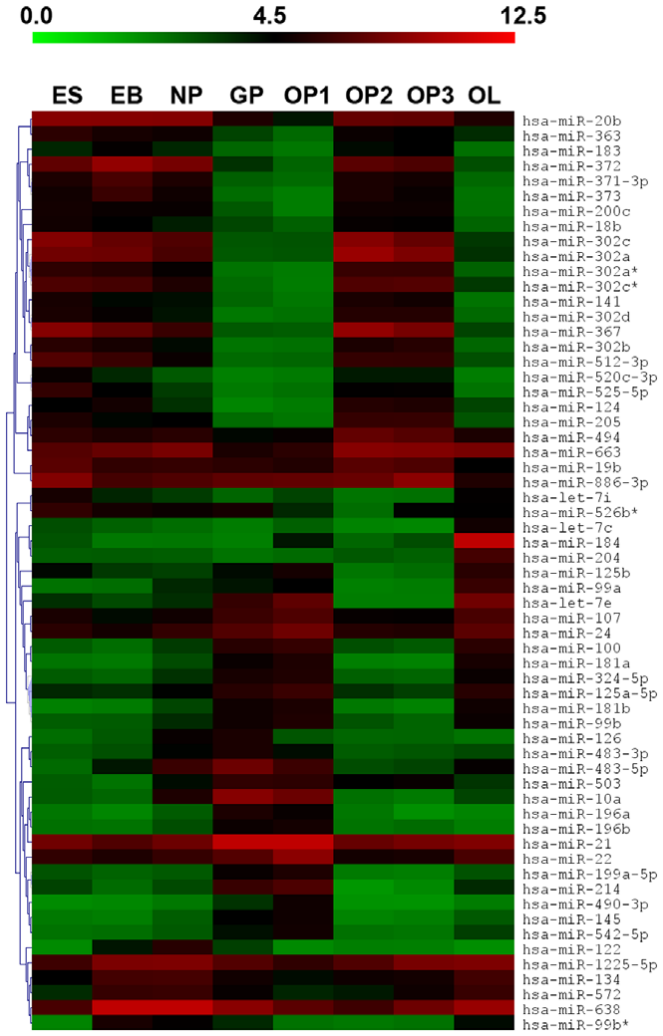
Hiereracheal clustering of top differentially expressed miRNAs. From the 183 detected miRNAs, those with variance in the top 30^th^ percentile across OL differentiation were selected for cluster analysis. Two prominent bimodal distributions resulted from this dataset.

Among the 866 miRNAs present on the array chip, 183 miRNAs were detected over eight stages of oligodendrocyte differentiation. The heat map in **Figure 2** shows the detected miRNAs displaying highest variance (top 30th percentile) throughout differentiation. From these miRNAs, two general bimodal patterns were observed across the eight stages. The expression of all detected miRNAs with greater than 2-fold expression between the eight stages is displayed in **Table S1**. The key miRNAs discussed in this manuscript were validated by conducting real-time qRT-PCR for samples from the appropriate stages, including the following: miR-199a and miR-145 at the OP1, OP2, OP3, and OL stages; miR-214 at the OP1 and OP2 stages; miR-184 and miR-1183 at the GP and OP1 stages (**Table 1**). The relative expression measured for these miRNAs show consistency with microarray results.

**Table 1:** Validation of key miRNAs.

	qPCR			microarray		
	FC mean	FC SE	p-value	FC mean	FC SE	p-value
**miR-199a-5p**						
OP1–OP2	−12.662	0.009	4.57E-04	−3.109	0.624	1.26E-01
OP2–OP3	−1.066	0.847	4.27E-01	−0.077	0.037	2.83E-01
OP3 - OL	13.959	1.893	8.58E-02	0.783	0.623	4.27E-01
**miR-145**						
OP1–OP2	−9.038	0.142	1.00E-02	−2.869	0.460	1.01E-01
OP2–OP3	0.239	0.121	2.98E-01	0.106	0.018	1.11E-01
OP3 - OL	8.381	2.511	1.85E-01	0.661	0.292	2.64E-01
**miR-214**						
OP1–OP2	−10.477	0.167	1.01E-02	−5.100	0.268	3.34E-02
**miR-184**						
GP - OP1	6.485	0.102	1.00E-02	1.864	0.174	5.94E-02
**miR-1183**						
GP - OP1	1.677	0.173	6.54E-02	2.130	-	-

Real-time qRT-PCR validation of microarray data for key miRNAs discussed in the text at appropriate stage transitions. Fold change means (log2), standard errors and p-values are listed for the two biological replications.

**Figure 3:**
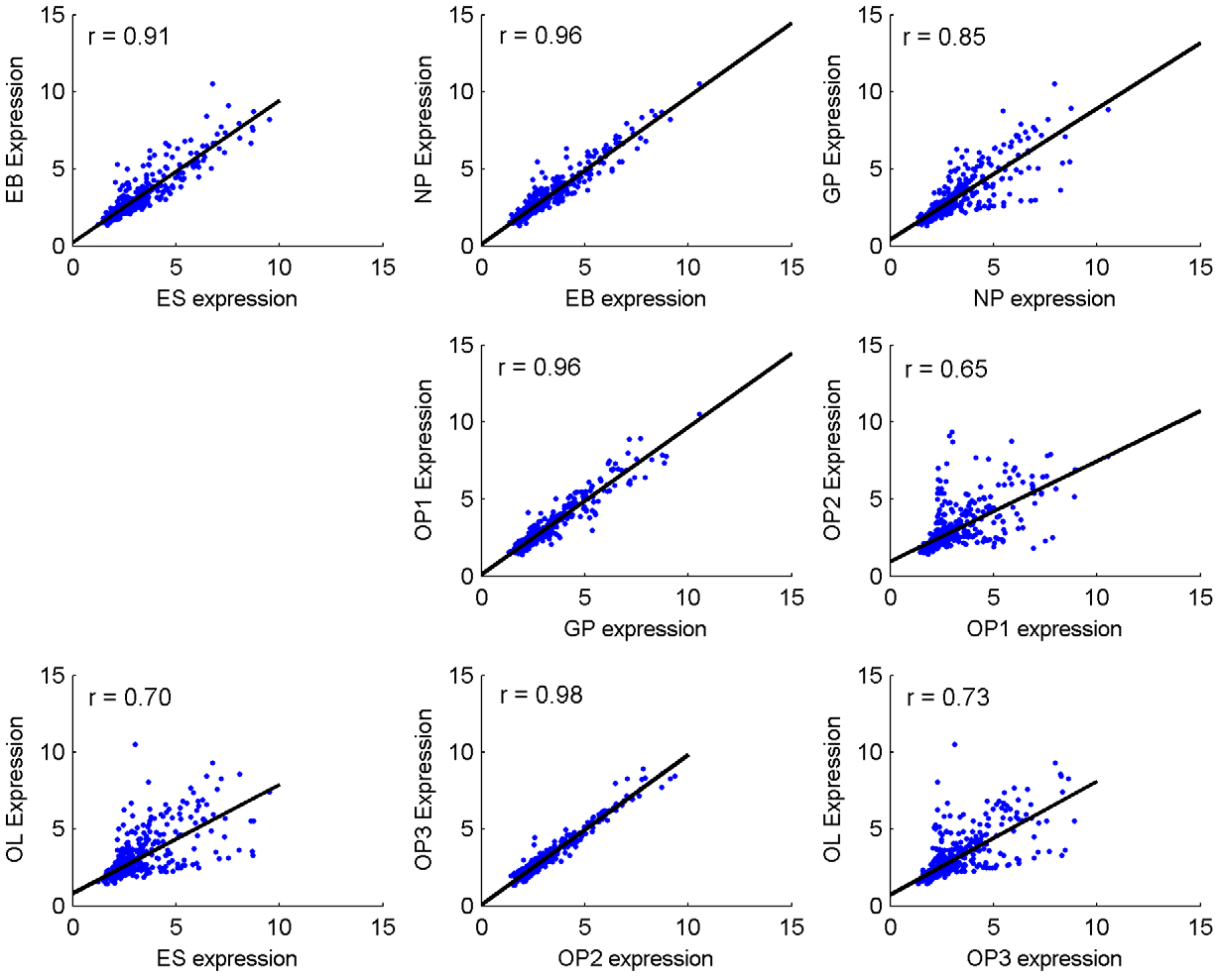
MiRNA correlation between stages. Correlation of miRNA expression levels (log2) between the ES, EB, NP, GP, OP1, OP2, OP3, and OL stages. Samples at each stage transition as well as the first (ES) and last (OL) stages of differentiation were compared. From the seven adjacent stage transitions, the OP2 to OP3 stages displayed the highest correlation (r = 0.98), while the OP1 to OP2 stages showed the least correlation (r = 0.65).

**Figure 4:**
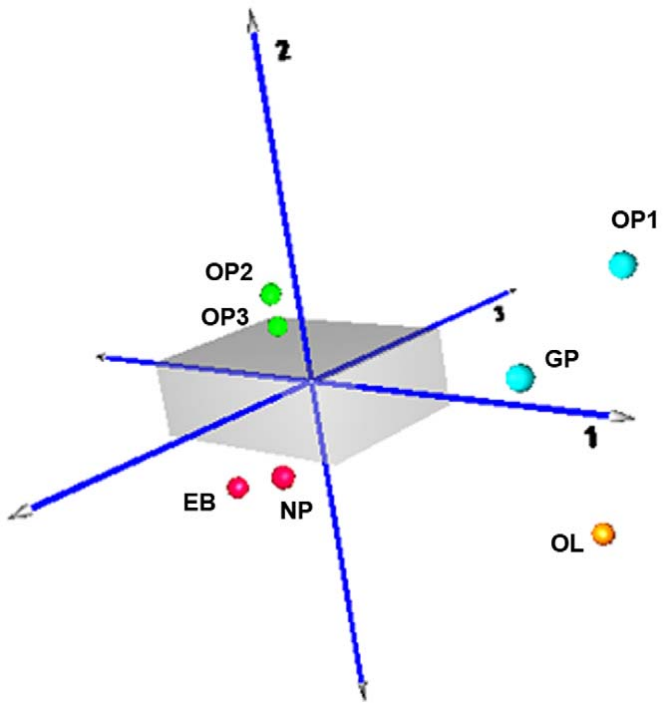
PCA analysis of OL differentiation. Principal components 1, 2, and 3 in 3D space, representing approximately 70% of the variation. Four main clusters were observed among the differentiating cells, including: (1) early neural progenitors (red), early glial progenitors (blue), (3) developing oligodendrocyte progenitors (green), and (4) mature oligodendrocytes (orange).

Using correlation analysis, we compared the overall miRNA expression profiles by studying the closest and furthest time points of differentiation (EB and OL, respectively). As expected, the overall miRNA expression levels of undifferentiated ES showed significantly lower correlations with differentiated OLs than EBs. Linear regression plots (**Figure 3**) of undifferentiated ES cells demonstrated much higher correlation to the EB stage (r = 0.91) than to the OL sage (r = 0.70). Another study demonstrated similar trends in ESCs, EBs and terminally differentiated cells during endothelial cell differentiation [32]. Our results demonstrate the importance of studying the lineage development of a cell type from ES cells versus just an undifferentiated and mature, or “very differentiated” state. For example, the cluster of miR-512-3p, miR-205, and the miR-302 family illustrated on the heat map demonstrates high expression in undifferentiated ES and early neural progenitor stages, downregulation during the glial restricted and early OP transitions, but then additional high expression during mid to late OP development. One possible explanation for this kind of expression patterning is that the miRNAs regulate different targets at different stages of OL development. Another is the result of targeting proteins with pleiotropic effects in different niches. Thus, our report emphasizes the complexity of studying miRNAs during differentiation by showing how certain miRNAs may have unrelated roles at various stages of lineage development. Principal component analysis (PCA) was conducted to detect overall variation between the different groups (**Figure 4**), which produced four main clusters among the differentiating cells: (1) early neural progenitors (2) early glial progenitors (GP and OL1 stages), (3) developing oligodendrocyte progenitors (OP2 and OP3 stages), and (4) mature oligodendrocytes (OL stage).

### MiRNAs demonstrating the highest differential expression at each stage-specific transition

**Figure 5:**
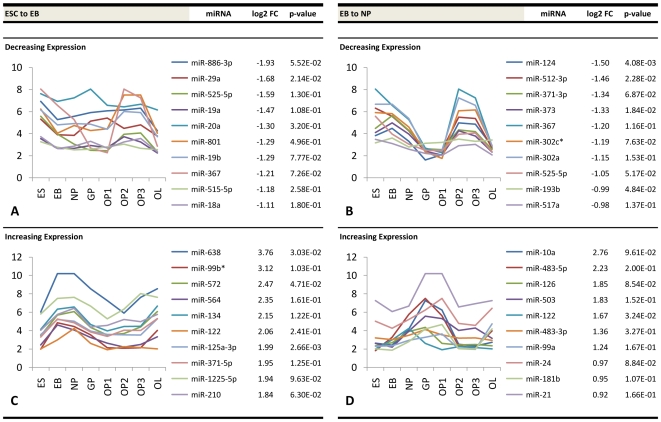
Top differentially expressed miRNAs at early neural progenitor stage transitions. Fold change values (log2) and expression profiles of the top ten downregulated miRNAs at the (a) ES-EB and (b) EB-NP stage transitions and the top ten corresponding upregulated miRNAs at the (c) ES-EB and (d) EB-NP stage transitions.

Our results show dynamic trends in miRNA expression during OL differentiation strongly suggesting, as it has in other systems, that miRNAs play an important role in regulating differentiation. Specifically, miRNAs which display significant changes at stage-specific transitions are likely to have key roles in regulating this process [10]. Thus, we focused our attention to miRNAs which demonstrated the greatest positive and negative fold changes at each stage transition. **Figure 5** summarizes the top ten increasing and decreasing miRNAs at early stage transitions of oligodendrocyte differentiation. At the ESC-EB stage transition, the top 10 miRNAs showing the greatest reduction in expression experienced a ∼3.8-fold change in expression. Similarly, the EB-NP stage transition showed modest downregulation, with a maximum ∼2.8-fold repression. Conversely, the top differentially upregulated miRNAs at these two stage transitions showed much greater changes, with a maximum ∼14-fold change at the ESC-EB transition and a maximum ∼6.8-fold change at the EB-NP transition.

**Figure 6:**
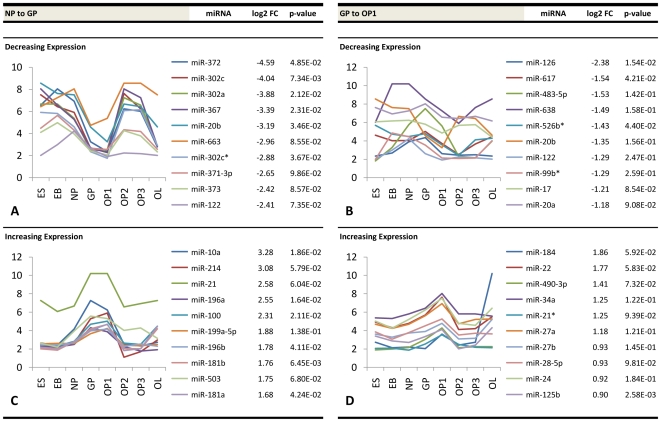
Top differentially expressed miRNAs at early glial precursor stage transitions. Fold change values (log2) and expression profiles of the top ten downregulated miRNAs at the (a) NP-GP and (b) GP-OP1 stage transitions and the top ten corresponding upregulated miRNAs at the (c) NP-GP and (d) GP-OP1 stage transitions.

**Figure 7:**
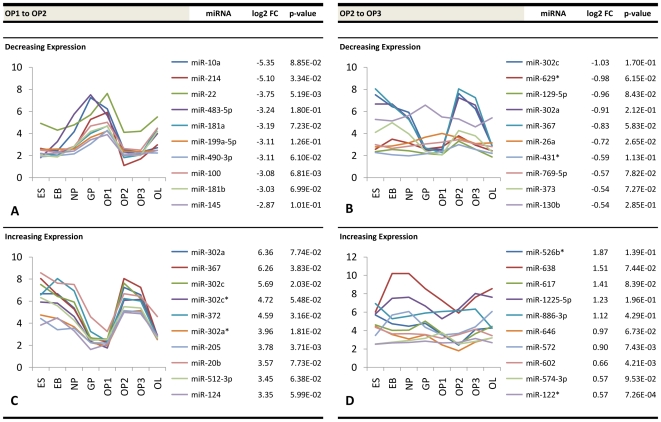
Top differentially expressed miRNAs at developing oligodendrocyte precursor stage transitions. Fold change values (log2) and expression profiles of the top ten downregulated miRNAs at the (a) OP1–OP2 and (b) OP2–OP3 stage transitions and the top ten corresponding upregulated miRNAs at the (c) OP1–OP2 and (d) OP2–OP3 stage transitions.

**Figure 8:**
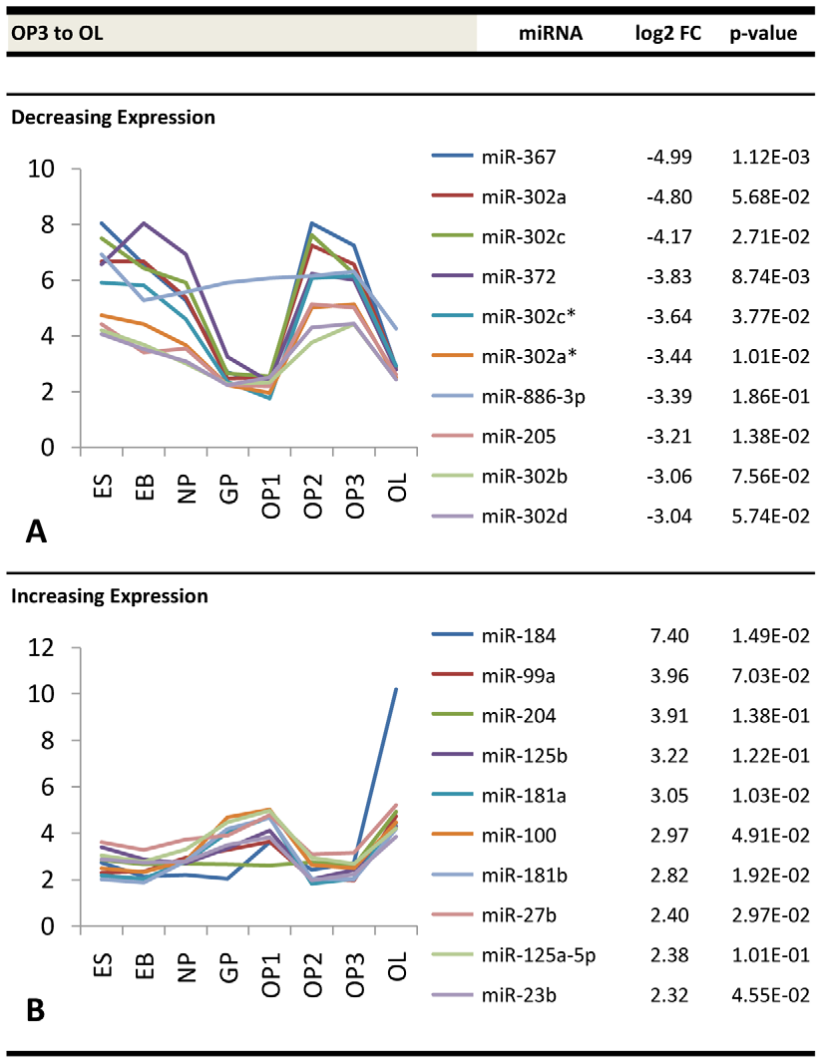
Top differentially expressed miRNAs at the late oligodendrocyte stage transition. Fold change values (log2) and expression profiles of the top ten downregulated miRNAs at the (a) OP3-OL stage transitions and the top ten corresponding upregulated miRNAs at the (b) OP3-OL stage transitions.

Similar information is presented for early glial progenitor stage transitions (NP to GP, GP to OP1) (**Figure 6**), developing oligodendrocyte precursor stage transitions (OP1 to OP2, OP2 to OP3) (**Figure 7**), and transition into the late oligodendrocyte stage (OP3 to OL) (**Figure 8**). The NP-GP transition showed steep negative differential expression with a maximum ∼24-fold change and high upregulation with a maximum ∼9.7-fold change. The transition from GP to OP1 was less pronounced in terms of both a maximum ∼5.2-fold downregulation and a maximum ∼3.6-fold upregulation. The most significant changes were seen at the OP1 to OP2 transition and at the OP3 to OL transition. The former stage showed a maximum ∼41-fold downregulation and maximum ∼6.4-fold upregulation, while the latter stage contained a maximum ∼32-fold positive change and a maximum ∼170-fold negative change. The OP2 and OP3 stages showed the least amount of change in miRNA expression, with a maximum ∼2.0-fold downregulation and ∼3.7-fold upregulation. The corresponding graphs within these figures plot the temporal expression of each of these differentially expressed miRNAs from the pluripotent ES stage to the differentiated OL stage. Unique trends within the accompanying plots show that groups of miRNAs with the highest fold changes at a given transition display a similar expression pattern during differentiation.

### Predicted protein targets corresponding to highly differentially expressed miRNAs

In addition to studying overall patterns of miRNA expression throughout oligodendrocyte differentiation, we identified several myelin-associated genes that have high target bias to the top differentially expressed miRNAs reported above (**Table 2**). These include chromosome 11 open reading frame 9 (***C11orf9*** *)*, myelin gene regulatory factor (***Mrf***), claudin-11 (***Cldn11*** *)*, myelin transcription factor 1-like (***Mytl1*** *)*, myelin-associated oligodendrocyte basic protein (***Mobp*** *)*, myelin protein zero-like 2 (***Mpzl2*** *)*, and discoidin domain receptor tyrosine kinase 1 (***Ddr1*** *)*. These target predictions were made by detecting miRNAs with significant expression changes at each step of differentiation between GPs and OLs.

**Table 2:** Predicted targets with referenced roles in myelination.

miRNA	gene symbol	gene name	seed match	context score percentile
**OP3 to OL**				
hsa-miR-205	CLDN11	claudin 11 (oligodendrocyte transmembrane protein)	8mer	97
hsa-miR-302a	ARHGEF10	Rho guanine nucleotide exchange factor (GEF) 10	7mer-m8	81
hsa-miR-302c	ARHGEF10	Rho guanine nucleotide exchange factor (GEF) 10	7mer-m8	81
hsa-miR-372	ARHGEF10	Rho guanine nucleotide exchange factor (GEF) 10	7mer-m8	93
hsa-miR-302b	ARHGEF10	Rho guanine nucleotide exchange factor (GEF) 10	7mer-m8	81
hsa-miR-302d	ARHGEF10	Rho guanine nucleotide exchange factor (GEF) 10	7mer-m8	81
**OP1 to OP2**				
hsa-miR-214	MOBP	myelin-associated oligodendrocyte basic protein	8mer	98
hsa-miR-199a-5p	C11orf9	chromosome 11 open reading frame 9	8mer	89
hsa-miR-199a-5p	C11orf9	chromosome 11 open reading frame 9	8mer	92
hsa-miR-199a-5p	C11orf9	chromosome 11 open reading frame 9	8mer	91
hsa-miR-199a-5p	DDR1	discoidin domain receptor tyrosine kinase 1	8mer	84
hsa-miR-199a-5p	DDR1	discoidin domain receptor tyrosine kinase 1	7mer-1A	4
hsa-miR-199a-5p	DDR1	discoidin domain receptor tyrosine kinase 1	8mer	70
hsa-miR-199a-5p	DDR1	discoidin domain receptor tyrosine kinase 1	8mer	69
hsa-miR-145	MPZL2	myelin protein zero-like 2	8mer	96
hsa-miR-145	MPZL2	myelin protein zero-like 2	7mer-m8	85
hsa-miR-145	QKI	quaking homolog, KH domain RNA binding	8mer	92
hsa-miR-145	C11orf9	chromosome 11 open reading frame 9	8mer	88
hsa-miR-145	C11orf9	chromosome 11 open reading frame 9	8mer	79
hsa-miR-181a	S1PR1	sphingosine-1-phosphate receptor 1	8mer	98

Predicted miRNA-mRNA interactions with stringent base pairing and conserved sites for the top negatively differentially regulated miRNAs during later stages of oligodendrocyte differentiation are listed. The seed match classification and context score percentile from the TargetScan database are reported for each predicted interaction. The OP1–OP2 and OP3-OL stage transitions were selected for their highly differential expression.

From our top ten differentially expressed miRNAs at the early OP to mid OP stage transition, two miRNAs (miR-199a-5p and miR-145) show strong target bias towards *C11Orf9*. This gene is speculated to be the human analog to the mouse MRF, a protein which has been shown to be critical for oligodendrocyte maturation and myelin production [33]. MiR-199a-5p has three sites with evolutionarily conserved 8mer seed matches to the 3′ UTR of *C11Orf9*, suggesting a high probability for miRNA-mRNA interactions. Importantly, miR-199a-5p showed a sharp ∼8.6-fold decrease in expression from the early OP to mid OP stage, followed by minimal ∼1.1-fold change during the mid OP to late OP stage transition. Then, miR-199a-5p levels rose (∼1.7 fold) at the final stage of differentiation. This miRNA expression pattern is in accordance with published MRF expression results, potentially suggesting that changes in miR-199a-5p expression may be concomitant with the initiation of MBP expression. However, while the increase of miR-199a-5p at the final stage corresponds with the decreased MRF expression at this transition, additional validation is necessary. In addition, miR-145 also followed a similar expression pattern increasing during the first transition from OP1 to OP2, stable during OP2 to OP3 and then subsequently increasing from OP3 to OL. MiR-199a-5p, with a ∼8.6-fold decrease from OP1 to OP2 stage, contains four total evolutionarily conserved target sites (three 8mer, one 7mer-1A) within the 3′ UTR of *DDR1* mRNA. Like MRF, DDR1 has been shown to be upregulated during oligodendrocyte differentiation and remyelination [34]. MiR-214 also shows a ∼34-fold decrease from the OP1 to OP2 transition and has a strong evolutionarily conserved 8mer target site to *Mobp*, which is important for providing the proper structural properties of myelin. Additionally, miR-205 showed a ∼9.2-fold downregulation during the OP3 to OL transition, which contains a conserved 8mer complementary site within the *Cldn11* 3′-UTR. This gene is expressed in oligodendrocytes, encoding for a transmembrane protein involved in interactions at tight junctions, and is the third most abundant protein in CNS myelin [35].

As GPs have the potential to derive both OLs and astrocytes, we studied targets that not only facilitate OP fate but also those that may inhibit astrocyte differentiation. By comparing GPs and OPs, we identified several miRNAs that inhibit targets that facilitate astrocyte differentiation. The highest upregulated miRNA (miR-184) at the GP-early OP transition shows a strong conserved 8mer binding site to BCL2-like 1 (***Bcl2l1***), a gene highly expressed in astrocytes [36]. MiR-1183 was also found to contain a stringent conserved binding site to the mRNA 3′ UTR of eukaryotic translation initiation factor 2B, subunit 5 epsilon (***Eif2b5***), which has been shown to facilitate astrocyte differentiation [37].

## Discussion

MiRNA expression profiling utilizing microarrays is a widely utilized procedure for the discovery of miRNA-mRNA interactions that regulate cellular processes. Most studies employ expression profiling as a starting point for identifying highly differentially expressed miRNAs that regulate a protein of interest. Since these studies focus on characterizing miRNA-mediated protein regulation at a particular stage transition, miRNA expression information is typically confined to narrow time frames. The overall goal of this study was to characterize the miRNA signature of several distinct stages that are essential for OL generation.

Interestingly, we discovered that miRNAs sharing the highest fold changes at a given transition demonstrated similar expression patterns over time. One potential explanation for this observation alludes to the more recent miRNA-mRNA interaction model proposed by Bartel and Chen [10], [11], in which miRNA induction can be thought of as a combinatorial rheostat that fine-tunes protein levels rather than acting solely as a classical binary off-switch. In this model, the 3′-UTR of one target mRNA molecule may contain several sites where different miRNAs may bind. The effective repression is then partly a function of the number of different miRNAs that bind to a particular 3′-UTR. It is possible that some of the miRNAs demonstrating similar temporal expression patterns in Figure 5, Figure 6, Figure 7 and Figure 8 may be co-regulating a shared set of mRNA targets. For instance, miR-145 and miR-199a-5p within our data showed similar expression patterns throughout differentiation and both contain conserved 8mer predicted seed pairings to multiple sites within the 3′-UTR of *C11orf9*. Thus, both miRNAs may co-regulate *C11orf9* through multiple sites within the same UTR and stoichiometrically affect gene expression by the number of sites they bind. This has been proposed to explain the graded versus binary effects of regulation observed in some miRNA interactions [11]. Another potential explanation for these trends is that groups of miRNAs regulate different proteins within common pathways. For instance, a study analyzing miRNA expression profiles of ovarian adenocarcinomas demonstrated that two similarly expressed miRNAs (miR-9 and miR-223) regulate two independent targets of a common pathway involved in ovarian metastatis [38]. Nonetheless, further investigations will be required to determine the relationship of commonly expressed miRNAs in oligodendrocyte differentiation.

Although no comparable investigations have been performed on human cells, some interesting parallels were detected between miRNAs found in this study and those reported in mouse and rat OL cell types. A study by Lau, *et al.* [20] explored the relative abundance of miRNAs within isolated rat CNS OP cells, CNS OLs, and PNS Schwann cells. We compared our data of GP and OP stages corresponding to A2B5+ GalC− and A2B5− GalC+ cell types from this study. From the top twenty miRNAs showing highest expression in A2B5+ GalC− cells, miR-130a, miR-16, miR-17, and miR-20a were also in the top twenty expressed miRNAs from our GPs. Similarly, miR-17, miR-20a, miR-21, miR-16, miR-103, and miR-107 identified in A2B5-GalC+ cells showed overlapping expression with our OPs. It is intuitive that the most highly expressed miRNAs at these stages may be potentially repressing the expression of astrocyte proteins. Interestingly, miR-16 was found to have an evolutionarily conserved 8mer target site with a high prediction score to GFAP, one of the most widely used markers for characterizing astrocytes. This same miRNA was also found to have a target bias for excitatory amino acid transporter 2 (***Eaat2*** *)*, which is upregulated in astrocyte progenitors [39]. Additionally, miR-17 and miR-20a were predicted to target membrane associated guanylate kinase, WW and PDZ domain containing 3 (***MagI-3***), a junctional protein found in astrocytes [40]. Finally, miR-21 was found to contain a strong target bias towards Fas ligand (***Faslg***), which is co-expressed with GFAP in astrocytes [41]. Another report demonstrates that miR-23 facilitates OL development by negatively regulating lamin B1 (**LMNB1**), a protein found to repress production of MBP, proteolipid protein 1 (**PLP**), and myelin oligodendrocyte glycoprotein (**MOG**) [19]. Expression data from our microarray results show high levels of miR-23a throughout the GP to OL stages. MiR-23b was also present in high levels during these same stages, although with reduced expression at the OP2 and OP3 stages.

In addition to miRNAs that have been previously reported, we also evaluated the predicted targets of the most differentially expressed miRNAs in our data with known or putative associations with oligodendrocyte differentiation and myelin production. One interesting predicted target is C11Orf9, which shows 80% homology to myelin gene regulatory factor (MRF) found in mice [33]. MRF is a recently discovered transcriptional regulator that is required for CNS myelination. RNAi knockdown experiments of MRF in cultured oligodendrocytic cells were found to prevent expression of many important CNS myelin genes, including CNPase, PLP, MAG, and MOBP. Conversely, overexpression of MRF within OP cells promoted myelin-associated gene expression. Mice without MRF could only generate premyelinating oligodendrocytes, which lacked myelin gene expression and failed to myelinate axons, eventually dying during the third postnatal week from severe neurological abnormalities. Under standard developmental conditions, MRF expression levels are low in early OP cells and significantly increase with OP cell maturation. As these cells mature into MOG+ OLs, MRF levels begin to decrease. Since miRNAs act by repressing gene expression, those targeting MRF would show inverse expression to the above trend. We tested, for the first time in human OLs, the expression of the human homolog to MRF, C11Orf9, which was detected at high levels in our immunofluorescence data. Target predictions for miR-199a-5p showed high likelihood for *C11Orf9* repressive interactions. Interestingly, miR-199a-5p demonstrated a large decrease in expression beginning at the early OP stage and an increase at the final stage. This would suggest a significant release of miRNA-mediated *C11Orf9* repression through OP cell differentiation, followed by a slight increase in repression at the terminal stage transition, in agreement with published mouse MRF results. Similarly, the expression pattern of miR-145 at these stages conformed to the same trend as miR-199a-5p. Therefore, these data suggest that miR-199a-5p and miR-145 may be simultaneously regulating the human homolog of MRF.

Besides having a strong target bias for *C11Orf9*, miR-199a-5p also has a significant bias for targetting DDR1, a tyrosine kinase receptor found to be upregulated during *in vivo* remyelination and *in vitro* oligodendrocyte differentiation [34]. The highly differential expression of miR-199a-5p, along with its high number of stringent evolutionarily conserved target sites to DDR1 mRNA, suggests a likely regulatory interaction involved in oligodendrocyte differentiation. In a similar fashion, another highly differentially expressed miRNA (miR-214) which displays a highly complementary alignment within the 3′ UTR of MOBP mRNA demonstrates a decrease in OP cells then a sharp increase in OLs. MOBP is abundantly produced within the cytoplasm of OP cells and later localized to the processes of mature OLs, containing clusters of positively charged amino acid residues necessary for myelin compaction [42]. Upon demyelination, electron microscopy studies have shown that mice lacking MOBP had problems compacting newly produced myelin suggesting that it is necessary for the proper structure and function of myelinated axons.

During the final stage transition, miR-205 showed significant downregulation. This miRNA shows a strong target bias for the 3′-UTR mRNA of CLDN11, a tight junction protein which has been shown to increase in expression as oligodendrocyte progenitors develop into mature oligodendrocytes [35]. As such, the decrease of miR-205 may be partially responsible for the increased expression of *Cldn11* at this stage. Several other differentially expressed miRNAs at this final stage transition may have important roles in the terminal differentiation of oligodendrocytes. From the top ten downregulated miRNAs at this transition, six miRNAs (miR-367, miR-302a, miR-302c, miR-372, miR-302b, and miR-302d) have been shown to encourage proliferation and are highly expressed in undifferentiated cells and cancer stem cells [43], [44], [45], [46], [47], [48]. This suggests that their decreased expression between the OP3 to OL stage may be associated with the inhibition of proliferation and the promotion of maturation in developing oligodendrocytes. On the other hand, the top upregulated miRNAs at the OP3-OL transition included miRNAs (miR-181a, miR-181b, miR-125b, and miR-184) that are associated with decreased proliferation in maturing CNS cells and decreased malignancy in glioma stem cells [49], [50], [51], [52], [53], [54], [55]. Thus, their increased expression suggests potential roles in OL maturation and normal oligodendritic development.

Conversely, another study demonstrated that the absence of miR-206 was essential for OL differentiation from rat bipotential oligodendrocyte-type 2-astrocyte (**O-2A**) progenitor cells which have the capability of generating both oligodendrocytes and astrocytes by targeting the tubilin polymerization-promoting protein (**TPPP/p25**), a marker of myelinating oligodendrocytes [21]. Our results also demonstrate a progressive decrease in miR-206 during ESC-derived OL differentiation. However, our data show a significant increase in this miRNA during the OP3-OL transition. There are several possible explanations for this increase. For instance, TPPP/p25 is a potential target for several miRNAs, including miR-1. Thus, an increase in one miRNA such as miR-206 may not be sufficient to significantly repress the expression of this protein. This is consistent with our results that suggest groups of miRNAs rather than a single miRNA may potentially regulate a common target. It is also not known whether these results are due to differences in rat and human, or whether the absence of miRNA-206 is only required for the initiation of the TPPP/p25 pathway prior to myelination. Similarly, recent rodent studies demonstrated the roles of miR-219[56], [57] and miR-338[57] in controlling oligodendrocyte differentiation. While our data showed many similarities between previous rodent studies, these two miRNAs were not detected in our human OP cells and OLs, signifying the importance of studying oligodendrocytes derived from human cells in addition to animal models.

Finally, we identified miRNAs with potential roles in preventing astrocytic differentiation at the point of astrocyte-oligodendrocyte lineage divergence (GP to early OP transition). The highest upregulated miRNA at this transition, miR-184, shows a high likelihood of binding to *Bcl2l1*, a gene which has been shown to be co-expressed with GFAP in various samples of astrocyte tissue [36]. In this study, BCL2L1 protein levels were found to be much higher than GFAP, suggesting a potentially strong role for this gene in astrocytes. Additionally, another positive differentially expressed miRNA at the GP-early OP transition, miR-1183, generated a high prediction score to an important enhancer of astrocyte differentiation, *Eif2b5*. Mutations in this gene have been associated with vanishing white matter disease [37]. While this leukodystrophy results in demyelination, GPs cultured with this mutation were able to produce normal OLs in culture. However, GPs with a mutation in *Eif2b5* produced very few numbers of GFAP+ astrocytes. Furthermore, *Eif2b5* RNAi studies also showed significantly decreased production of GFAP+ astrocytes from human fetal GPs. Therefore, it is possible that transfection of miR-184 and/or miR-1183 analogs to GPs may help reduce astrocyte differentiation and promote the production of oligodendrocyte cultures with increased purity.

In summary, we have shown that our analyses of miRNA profiling can be used to distinguish between different stages of oligodendrocyte differentiation. Specifically, we have identified a number of miRNAs which may potentially regulate or mark key steps in OL differentiation from human ES cells, along with their potential protein targets. These interactions may have significant roles in oligodendrocyte differentiation and myelin production.

## Materials and Methods

### Cell culture

H1 (WA01) purchased from WiCell (Madison, WI) and expanded in ESC growth media^1^ and differentiated according to modified published protocols^2^ into embryoid bodies (EBs), neural progenitors (NPs), glial restricted precursors (GPs), oligodendrocyte precursors (OPs), and finally premyelinating oligodendrocytes (OLs). Neural differentiation of ESCs were initiated via embryoid body (EB) suspensions with B27 medium [58] supplemented with 200 ng/ml noggin, 20 ng/ml FGF2, and 20 ng/ml FGF4 (all from R&D Systems). EBs (or early neural progenitors) were grown for 15 days and then plated on matrigel and grown in B27 media supplemented with 20 ng/ml FGF2 for 5 days producing late neural progenitors (**NP**s), which were identified by A2B5 expression. Neural progenitors were isolated by magnetic-activated cell sorting (MACs) by A2B5 which produced >99% A2B5+ and Nestin+ cells.

Populations of glial progenitors (**GPs**) which are capable of generating both astrocytes and oligodendrocytes were distinguished from neural progenitor cultures by their expression of PDGFRα and Olig1, and distinguished from more differentiated OP cells by their lack of expression of O1 and O4. Cultures containing GP cells were then split onto matrigel with 0.05% trypsin/EDTA and further differentiated into oligodendrocyte progenitors in B27 media with the addition of 20 ng/ml of PDGF (PeproTech) and 20 ng/ml EGF (R&D Systems) for 25 days, first expressing O4 (pre-oligodendrocytic progenitors) and later expressing O1, GalC and CNPase markers (pro-oligodendrocytic progenitors) Terminal differentiation into mature OLs was induced in B27 supplemented with 40 ng/ml 3,3′5-Triido-L-thyronine (T3) for an additional three weeks. Mature OLs were distinguished from OP cells by their expression of myelin basic protein. After the addition of the appropriate factors, GPs, OPs and OLs were isolated for analysis. For each stage, cell purity was based on ICC, as performed previously [24]. These studies showed at least 95% purity in our cultures based on marker expression. Importantly, OLs and not OPCs expressed MBP.

### Immunofluorescence

Immunostaining was performed on tissue and on cells using anti-Oligodendrocyte Specific Protein (CLDNanti-Oligodendrocyte marker 1 (O1, 1∶20, Millipore) mouse anti-Oligodendrocyte marker 4 (O4, 1∶20, Millipore), rabbit anti-galactocerebroside (GalC, 1∶20, Millipore), anti-A2B5 (1∶20, Millipore), mouse anti-nestin (1∶20, Millipore), rabbit anti-Pax6 (1∶20, Abcam), rabbit anti-NG2 (1∶20, Millipore), goat anti-Sox10 (1∶20, Santa Cruz), rabbit anti-PDGFRα (1∶20, Abcam), mouse anti-Olig1 (1∶20, Millipore), rat anti-MBP (1∶20, Abcam) and mouse anti-CNPase (1∶20, Millipore) using standard protocols along with fluorescent secondary antibodies (Millipore). DAPI was used to stain nuclei to determine the percentage of immunopositive cells.

### miRNA isolation

Eight total populations were isolated for miRNA analyses. These included undifferentiated ES cells, EB cells, NP cells, GP cells, OP cells (Day 7, Day 14, Day 25), and OL cells. MiRNA was isolated from ∼2×10^5^ cells using miRNeasy Mini kits (Qiagen) followed by quality checks of both total RNA and small RNA using a 2100 Bioanalyzer and software which detected 28S and 18S ribosomal RNA ratios, generated a RNA Integrity Number (RIN), concentration of sample, and ribosomal ratio. Only samples with 28S/18S>1.2, RIN>8 and detectable miRNA were used for the study.

### miRNA microarray

Human miRNA Microarray V3 kits (G4470C, Agilent Technologies) were employed for this study following manufacturer's protocols. The Agilent miRNArrays contain probes for all 866 human and 89 human viral miRNAs reported from the Sanger database v12.0 (http://microrna.sanger.ac.uk/sequences/). Each miRNA species is printed 20 times with replicate probes on the array. 100 ng of total RNA was dephosphorylated with 11.2 units of calf intestine alkaline phosphatase at 37°C for 30 minutes and followed by end-labeling with pCp-Cy3 (Agilent Technologies) and 15 units of T4 RNA ligase (GE Healthcare) at 16°C for 2 hours. Labeled samples were purified with Micro Bio-Spin 6 columns (Bio-Rad). Labeling efficiency and nucleic acid concentration was measured using Nanodrop 1000. Samples were mixed with 10x blocking agent and 2x Hi-RPM hybridization buffer (Agilent Technologies) and microarray hybridization was carried out at 55°C with rotation at 20 rpm in a designated Agilent G2545A hybridization oven for 20 hours. Microarrays were washed and scanned using an Agilent Scanner controlled by Agilent Scan Control 7.0 software. Hybridization images were scanned with Agilent Feature Extraction 9.5.3.1 software for miRNA microarray. Microarray analysis was performed for two biological replicates.

### RT-PCR validation

Quantitation of miRNAs was carried out using TaqMan® microRNA Assays (Applied Biosystems). Briefly, 3.34 ng of template total RNA was reverse-transcribed in a 5 µL reaction using the TaqMan® MicroRNA Reverse Transcription Kit and miRNA-specific stem-loop primers for candidate miRNA (Applied Biosystems). The reverse transcription reaction was diluted by adding 32.3 µL H_2_O, then 2.25 µL of the diluted mixture was introduced into a 5 µL PCR reaction using TaqMan® Universal PCR master mix without AmpErase UNG. Real-time PCR was performed using an Applied Biosystems 7900HT Thermocycler at 95°C for 10 min, followed by 40 cycles of 95°C for 15 sec and 60°C for 1 min. Real-time qRT-PCR was performed for two biological replicates with technical triplicates. The 18S rRNA was used as the housekeeping gene.

### miRNA analysis

After hybridization images were digitized using Agilent Feature Extraction 9.5.3.1 software, the average background intensity was computed and subtracted from each digitized raw intensity value. The software then computed the total error for each background-subtracted value. Detectable miRNAs were defined as those containing expression levels greater than three times the computed error for at least one stage. Detected background-subtracted data were converted to log2 values. The microarray data reported in this manuscript is described in accordance with MIAME guidelines. Quantile normalization was performed to account for differences in microarray input between samples. Pearson correlation coefficients were calculated for miRNA expression levels between each subsequent stage of oligodendrocyte differentiation. Fold changes at each of the seven stage transitions were computed for each miRNA and the greatest positive and negative results were ranked. Paired two-sample t-tests were carried for each of the top differentially expressed miRNAs at each stage transition. Corresponding p-values were derived from the theoretical t-distributions. The above data processing was carried out with custom algorithms scripted within MATLAB (Mathworks, Natick, MA). Filtering was utilized to select the miRNAs displaying the highest 30^th^ percentile variance throughout the eight stages of differentiation for cluster analysis. Hierarchal clustering using an implementation of average linkage and Euclidean distance metric was performed for these filtered miRNAs. TIGR MeV (Multiple Experimental Viewer) software [59] was utilized to create clustergrams as well as principal component analysis plots of the first three components of variation.

### miRNA target prediction

Targets of the top differentially expressed miRNAs at GP through OL stages were identified using several different sources of publicly-available software as each program uses its own unique algorithms to measure complementarity. To filter this extensive set of predicted targets, a custom algorithm was developed within MATLAB to retrieve prediction data from the TargetScan database [60] and conduct an automated Entrez Gene database search to only return proteins with reported roles in myelination and oligodendrocyte differentiation. This dataset was used as a basis for a focused literature search from the set of all proteins that display miRNA target bias. Similar targets were also confirmed with other prediction software that employ stringent base pairing and evolutionary conservation, including PicTar (http://pictar.bio.nyu.edu/) and EMBL (http://www.ebi.ac.uk/embl/).

## Supporting Information

Table S1 MiRNAs displaying >1.0 (log2) fold change at the seven stage transitions studied. Fold changes (±SEM) at each stage transition of oligodendrocyte differentiation are included.
(0.04 MB XLS)
